# siRNA‐Based Nanodrug Delivery Systems in the Treatment of Autoimmune Diseases

**DOI:** 10.1155/jimr/2307551

**Published:** 2026-08-02

**Authors:** Yi Liu, Zhihui Feng, Biao Zhang, Xueli Yang, Fei Yang, Guiya Lu, Chunhong Li, Chune Mo, Minglin Ou, Xianliang Hou

**Affiliations:** ^1^ Laboratory Center, Guangxi Key Laboratory of Metabolic Reprogramming and Intelligent Medical Engineering for Chronic Diseases, The Second Affiliated Hospital of Guilin Medical University, Guilin 541199, China, glmc.edu.cn; ^2^ Department of Central Laboratory, Shenzhen Hospital (Longgang), Beijing University of Chinese Medicine, Shenzhen 518172, China, bucm.edu.cn

**Keywords:** autoimmune diseases, nanomaterials, rheumatoid arthritis, RNAi, siRNA

## Abstract

Autoimmune diseases are chronic disorders with complex pathogenesis. Immune homeostasis disruption is closely associated with disease initiation and progression. Despite extensive research, the precise mechanisms underlying these diseases remain elusive. Conventional therapeutic approaches for autoimmune diseases primarily involve the administration of immunosuppressants, glucocorticoids, and monoclonal antibodies, each of which presents certain limitations. The emergence of RNA interference (RNAi) therapeutics has introduced a novel avenue for addressing these conditions. The development of an optimally engineered vector for RNAi drugs is anticipated to significantly enhance their clinical applicability. Nanomaterials, as promising drug carriers, offer robust support for RNAi therapeutics. This review summarizes recent advances and applications of nanodelivery systems for small interfering RNA (siRNA) therapeutics in autoimmune disease treatment. It also analyzes the challenges and future prospects of nanomaterial application in this field.

## 1. Introduction

Autoimmune diseases primarily result from aberrant immune responses against endogenous substances and tissues and are classified as a heterogeneous group of “common complex disorders.” Both genetic and environmental factors significantly contribute to the etiopathogenesis of these diseases. Most autoimmune diseases have a prevalence ranging from 0.1% to 1.0% in the general population [1]. The workings of the RNA interference (RNAi) system were first understood in 1998 [2]. Small interfering RNAs (siRNAs) induce RNAi via sequence‐specific degradation of target mRNAs, efficiently silencing genes [3]. Leveraging this mechanistic specificity, RNAi‐based therapeutics have garnered substantial attention as a promising modality for the intervention of autoimmune diseases. This review first summarizes the biological characteristics and therapeutic advantages of siRNA. It then illustrates the research progress of siRNA‐loaded nanodelivery systems for rheumatoid arthritis (RA), focusing on macrophages, key signaling pathways, and B cells. Furthermore, the current applications of such nanoplatforms in other prototypical autoimmune disorders, including inflammatory bowel disease (IBD) and psoriasis, are briefly recapitulated. This article highlights the major challenges of RNAi therapeutics, including off‐target effects and inherent immunogenicity. It also prospects future research directions, aiming to provide theoretical and practical references for developing precise targeted therapies against autoimmune diseases.

## 2. siRNA

**Figure 1:**
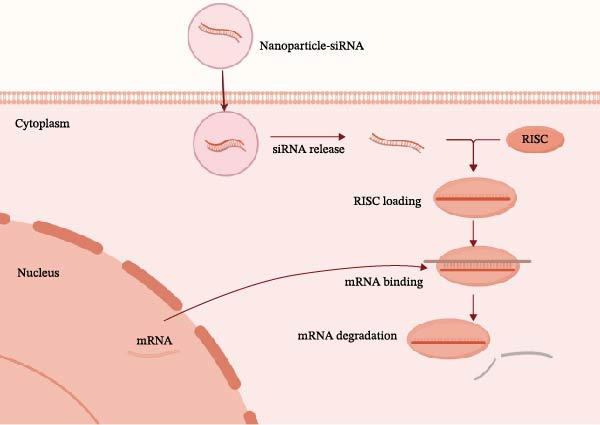
The process involves several key steps: (1) the introduction of small siRNA into the cell via an appropriate nanocarrier; (2) the release of siRNA by nanomaterials; (3) the binding of siRNA to the ribonucleoprotein complex RISC within the cytoplasm, followed by the degradation of the siRNA passenger strand; and (4) the activated RISC identifies the complementary mRNA of the target gene, resulting in mRNA degradation.

Within the RNAi pathway, multiple RNA species are active, with siRNA serving as a crucial effector molecule. Although siRNA holds great potential for drug development, its broad clinical use is limited by various intracellular and extracellular obstacles. The delivery of unmodified and unprotected siRNA faces multiple challenges, such as off‐target effects and immunogenic responses. Moreover, naked siRNA is easily degraded by nucleases in biological fluids, leading to poor stability, low bioavailability, and reduced therapeutic efficacy [4]. Consequently, there is an ongoing pursuit to identify optimal delivery systems for siRNA to ensure precise targeting. In recent times, nanoparticles (NPs) have become a potential delivery system for RNAi treatments. They have shown the ability to effectively transport siRNA while reducing toxicity and minimizing unintended effects. RNAi‐based therapeutics have gained clinical validation across a range of diseases, with several siRNA drugs obtaining regulatory approval for targeted disease modulation. A good example is patisiran (Onpattro), an siRNA therapy approved by the FDA. It targets transthyretin (TTR) mRNA and is the first RNAi‐based drug for treating hereditary ATTR amyloidosis [5]. siRNA is a double‐stranded molecule made up of 21–23 nucleotides. It enables specific gene silencing by guiding the RISC to degrade mRNA. This siRNA duplex is generated when long double‐stranded RNA is cleaved by Dicer, an enzyme belonging to the RNAse III family. The duplex comprises a passenger strand and a guide strand, with the latter serving as the antisense strand. The siRNA joins the RISC, interacting with the Argonaute 2 protein. This causes the double‐stranded siRNA to separate, and the passenger strand is degraded. The guide strand in the RISC then pairs with matching mRNA, forming a RISC‐mRNA complex, which ultimately leads to gene silencing (Figure 1) [6, 7].

**Figure 2:**
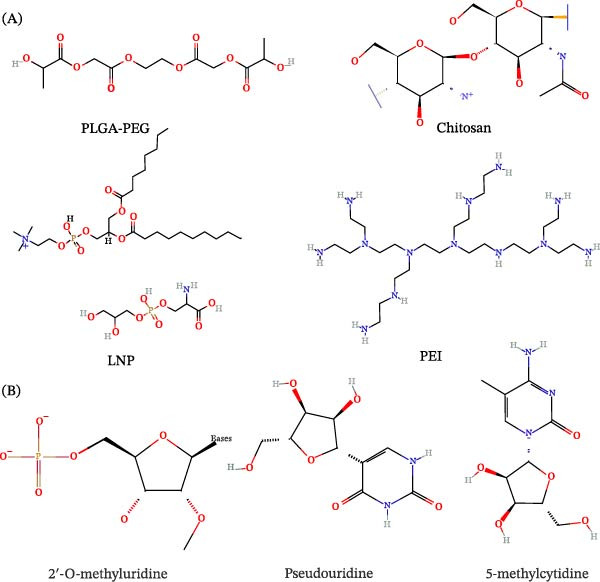
Chemical structures of representative nanocarriers and siRNA chemical modifications. (A) Poly(lactide‐co‐glycolide)‐block‐poly(ethylene glycol) (PLGA‐PEG), chitosan, lipid nanoparticle (LNP), and polyethyleneimine (PEI). (B) Common siRNA chemical modifications: 2′‐O‐methyluridine, pseudouridine, and 5‐methylcytidine.

To improve the stability, reduce immunogenicity, and facilitate efficient delivery of siRNA, various nanocarriers and chemical modification strategies have been widely developed, as summarized in Figure 2.

## 3. Nano‐siRNA for RA

RA, the most common autoimmune form of arthritis, affects ~1% of the population [8]. RA is characterized by persistent synovitis, cartilage damage, and joint deformity. Macrophages, signaling pathways, and B cells play critical roles in RA pathogenesis [9]. Thus, these components might serve as targets for RA treatment. Developing drugs with siRNA that focus on macrophages, signaling pathways, or B cells provides fresh possibilities for RA clinical management, addressing hurdles that are hard to surmount with standard surgical or medical techniques.

### 3.1. Targeting Macrophages

Macrophages, known for their high plasticity, are innate immune cells that can produce a wide range of inflammatory cytokines. As a result, these cytokines help attract and recruit various immune cells, including macrophages. A heterogeneous group of immune cells, macrophages critically orchestrate the immune response in RA [10]. Consequently, focusing on macrophages has been recognized as a promising therapeutic approach.

MCL‐1, an antiapoptotic Bcl‐2 family protein, represents a potential therapeutic target for RA [11]. Macrophages primarily secrete various proinflammatory cytokines, including interleukin‐1β, serum interleukin‐15, and TNF‐α. These cytokines can activate multiple immune cells, trigger an inflammatory cascade, and eventually lead to cartilage damage and bone erosion [12–14]. Given this understanding, researchers have focused on macrophages and developed siRNAs targeting MCL‐1, TNF‐α, IL‐2/15Rb, and IL‐1β, among others, to investigate their potential therapeutic benefits for RA.

#### 3.1.1. MCL‐1 siRNA

MCL‐1, a tightly regulated member of the B‐cell lymphoma 2 family, is reported to be overexpressed in macrophages within arthritic joints and is crucial for their survival [15, 16]. MCL‐1 stops macrophages from dying by blocking the activation of proapoptotic markers such as Bax and Bim. Studies have shown that reducing MCL‐1 levels can trigger macrophage apoptosis, highlighting its significance in treating RA [17]. Relevant to this investigation, Li et al. [18] worked on creating folate‐modified PLGA‐based polymeric micelles to deliver both MCL‐1 siRNA and dexamethasone together. This strategy aims to improve therapeutic efficacy. These folate‐linked micelles loaded with DEX and siRNA were found to greatly reduce MCL‐1 mRNA expression and significantly lower TNF‐α and IL‐1β levels compared to the adjuvant‐induced arthritis model and untreated groups. This delivery system is seen as a potential way to effectively deliver combination therapies for RA.

#### 3.1.2. TNF‐α siRNA

TNF‐α, a key proinflammatory cytokine primarily secreted by macrophages, is significantly elevated in RA. This upregulation drives persistent inflammation, fosters an inflammatory microenvironment, and contributes to severe joint damage [19]. Given TNF‐α’s critical role in RA pathogenesis, therapeutic strategies targeting its inhibition hold significant promise for RA treatment [20]. In a study, Shi et al. [21] developed a modified chitosan nanocarrier containing folic acid, diethylethylamine, and polyethylene glycol (PEG) (folate‐PEGCH‐DEAE15). This nanocarrier protects siRNA from nuclease degradation and improves cellular uptake via ligand–receptor interaction. It also increases solubility at neutral pH and promotes payload delivery into target cells. Additionally, this siRNA nanocarrier effectively reduced cartilage damage and prevented bone loss. Liu et al. [22] engineered a biomimetic NP coated with macrophage plasma membrane and functionalized with DNA nanostructures to enable dual‐targeting inhibition of TNF‐α. This study demonstrates the significant therapeutic promise of siRNA as a safe, targeted, and effective RNAi‐based strategy for RA. More recently, Feng et al. [23] introduced an innovative lipid NP (LNP) platform for the codelivery of the small‐molecule drug hydroxychloroquine and siRNA targeting TNF‐α (siTNF‐α) for RA treatment. Their findings demonstrate that LNPs can effectively coencapsulate nucleic acids and small‐molecule drugs, serving as safe and efficient delivery vectors. This provides a promising combinatorial strategy for future RA drug development.

#### 3.1.3. IL‐2/15Rβ siRNA

IL‐15, a proinflammatory cytokine, plays a pleiotropic role in immune system regulation. Elevated IL‐15 levels in the serum and synovial fluid of RA patients—consistently observed in clinical studies—underscore its critical role in disease immunopathology and correlation with clinical disease activity [24, 25]. In the context of experimental arthritis, Zhang et al. [26] evaluated the therapeutic effects of delivering siRNAs targeting the IL‐15 receptor β chain, which is also part of the IL‐2 receptor. In a study involving rats with AA, the therapeutic impact of intravenous administration of siRNA targeting the IL‐15Rβ chain was investigated. A reduction in the expression of proinflammatory mediators within the inflamed joints was associated with the observed therapeutic effect. IL‐2/15Rβ siRNA combined with polyethylenimine can be efficiently internalized by inflamed macrophages. Duan et al. [27] synthesized and characterized polyethyleneimine (PEI)‐SPIO/siRNA‐NPs in vitro and then administered them intravenously to arthritic rats to assess cellular uptake, biodistribution, and therapeutic efficacy against the shared IL‐2/IL‐15Rβ. They demonstrated that PEI‐functionalized SPIO NPs enable systemic siRNA delivery for RA treatment, with therapeutic efficacy significantly enhanced by applying an external magnetic field.

#### 3.1.4. IL‐1β siRNA

IL‐1 family cytokines serve as pivotal mediators in modulating immune responses [28]. This family encompasses key proinflammatory mediators, including IL‐1α, IL‐1β, IL‐18, and IL‐33. IL‐1β–expressing macrophages are implicated in RA pathogenesis and proposed as key pathogenic drivers. Relevant to this investigation, Song et al. [29] showed that lipidoid‐polymer hybrid NPs can effectively transport siRNA targeting IL‐1β to macrophages. This delivery method significantly reduced the progression of experimental arthritis in collagen antibody–induced arthritis mice. The above macrophage‐targeting siRNA nanodelivery systems (e.g., MCL‐1, TNF‐α, IL‐2/15Rβ, and IL‐1β siRNAs) and their corresponding nanocarriers are summarized in Table 1.

**Table 1:** Nanoparticle‐based siRNA delivery systems for the treatment of autoimmune diseases.

Nanoparticle	siRNA	Targeting	Ref.	Diseases
Poly(lactic‐co‐glycolic) acid	MCL‐1	Macrophages	[18]	RA
Chitosan	TNF‐α	Macrophages	[21]	RA
LNP	TNF‐α	Macrophages	[22]	RA
LNP	TNF‐α	Macrophages	[23]	RA
PEI	IL‐2/15Rb	Macrophages	[27]	RA
LNP	IL‐1β	Macrophages	[29]	RA
LNP	p65	Notch pathways	[34]	RA
Chitosan	Notch1	Notch pathways	[39]	RA
PEG‐PLGA‐DOTAP	BAFF‐R	B cells	[41]	RA
PLA‐PEG‐maleimide‐Ab	TNF‐α	Macrophages	[44]	IBD
Polymer‐lipid nanoparticles	TNF‐α	Macrophages	[46]	Psoriasis

### 3.2. Targeting Notch Pathways

In RA, the Notch signaling pathway plays a crucial role in synoviocytes. It regulates osteoclast differentiation and bone resorption by directly impacting osteoclast precursors and indirectly influencing cells in the osteoblast lineage and the immune system [30]. RA is marked by the overactivation of the NF‐kB signaling pathway. Therefore, blocking this pathway is an important way to control the inflammation related to RA [31, 32]. In the NF‐kB family, p65 is a key player in regulating inflammation. At the same time, the overactivation of Notch1 signaling in synovial tissue is involved in causing joint damage. Consequently, the development of p65 siRNA and Notch1 siRNA targeting the Notch pathways presents a promising therapeutic approach for RA.

#### 3.2.1. p65 siRNA

The p65 protein possesses transcriptional activation capabilities; upon activation of the classical pathway, p65 forms heterodimers with p50 or other subunits. These p50/p65 dimers attach to DNA sequences, triggering the transcription of many target genes and controlling the expression of proinflammatory factors. This helps reduce the inflammatory process [33]. Relevant to this investigation, Wang et al. [34] selected p65 siRNA, which directly targets the NF‐kB family member p65, potentially inhibiting NF‐kB–mediated inflammatory activities. It was demonstrated that the combination of siRNA and methotrexate in a calcium phosphate/liposome–based hybrid nanocarrier could be an effective treatment for RA. Their study showed that the folate receptor–targeted nanocarrier system significantly slowed down the progression of arthritis in mice. NF‐kB signaling pathways were effectively obstructed by combinational NPs [35].

#### 3.2.2. Notch1 siRNA

Notch1 is a signaling receptor involved in many cellular processes, such as development, differentiation, proliferation, survival, and apoptosis [36, 37]. Jong‐Sung Park’s recent research has shown that blocking Notch1 activation can reduce the severity of inflammatory arthritis. It also suppresses NF‐kB, proinflammatory cytokines (like TNF, IFN‐γ, MCP‐1, IL‐6, IL‐12, and IL‐17), and matrix metalloproteinase‐3 in CIA mice and RA synoviocytes [38]. To validate the anti‐inflammatory effects in the CIA model, Kim et al. [39] developed Notch1‐targeting siRNA delivery NPs. These siRNA‐NPs were created by packing polymerized siRNA into thiolated glycol chitosan NPs in water. The ability of siRNA‐NPs to block Notch1 in mouse macrophage cells (RAW 264.7) was proven using confocal microscopy and real‐time PCR. Also, fluorescently tagged siRNA‐NPs were successfully introduced into RAW 264.7 cells, changing the Notch1 expression at the mRNA level. A novel delivery system utilizing siRNA‐NPs targeting Notch1 has demonstrated effective treatment of RA by suppressing the Notch1 signaling pathway while avoiding significant toxicity. This method shows that Notch1‐inhibiting siRNA‐NPs have significant potential for treating RA, which is hard to reach with traditional drugs. The nanodelivery systems targeting the Notch signaling pathway (p65 and Notch1 siRNAs) are also summarized in Table 1.

### 3.3. Targeting B Cells

The development of RA is closely linked to B cells because of their roles in presenting antigens, secreting cytokines, and producing autoantibodies. At the early stage of RA, B cells secrete cytokines and autoantibodies to regulate the activation and differentiation of synovial T cells, monocytes, and osteoclasts [40]. The B‐cell activating factor receptor (BAFF‐R) plays a crucial role in managing B‐cell functions, especially their maturation and survival. Wu et al. [41] effectively designed a NP containing siBAFF‐R to target B cells in vitro and in a collagen‐induced RA mouse model in vivo. By enhancing siRNA delivery into B cells in vivo, these NPs contributed to the alleviation of disease symptoms in mice with RA. The way NPsiBAFF‐R works in RA includes reducing the percentage and number of B cells, as well as stopping inflammatory cytokines in the joints and blood. Based on these results, NPs carrying siBAFF‐R are a good way to treat RA by changing B cell numbers and reducing inflammation. The BAFF‐R siRNA nanodelivery system targeting B cells is likewise summarized in Table 1.

## 4. Nano‐siRNA for Other Autoimmune Diseases

### 4.1. Nano‐siRNA for IBD

IBD is a chronic inflammatory disorder of the gastrointestinal tract. Over recent decades, IBD incidence has risen markedly in both developed and developing countries, driven by factors including population growth and dietary shifts toward processed foods [42]. TNF‐α is mainly released by macrophages. During IBD, higher levels of TNF‐α can increase the production of proinflammatory cytokines like IL‐1, IL‐6, and IL‐8, which makes the inflammation worse at the site of the disease.

siRNAs targeting proinflammatory cytokines have garnered considerable interest as therapeutic candidates for intestinal inflammation, leveraging RNAi to silence pathogenic cytokine expression [43]. In contrast to traditional pharmacological treatments, RNAi therapy selectively targets intestinal tissues without inducing systemic immunosuppression, offering a stable and reliable approach for IBD management. Relevant to this investigation, Laroui et al. [44] looked into the potential of colon‐targeting NPs that can directly release specific siRNAs to target cells. Their research showed that orally given carriers targeting macrophages and loaded with siRNA can release TNF‐α siRNA directly into intestinal macrophages, reducing colitis. This suggests that poly(lactic acid)‐poly(ethylene glycol) (PLA‐PEG) NPs linked with Fab’ fragments are a powerful and efficient tool for delivering siRNA to colonic macrophages.

### 4.2. Nano‐siRNA for Psoriasis

Psoriasis is a long‐term inflammatory skin condition marked by high levels of TNF‐α, a proinflammatory cytokine. It ranks among the most prevalent skin disorders and poses ongoing challenges in terms of therapeutic management [45]. Currently, both topical and systemic treatments constitute the primary therapeutic strategies for psoriasis. However, these interventions often face limitations in long‐term application due to concerns regarding drug safety profiles and diminished efficacy, which impede effective disease control. Nevertheless, advancements in technology have facilitated innovations in therapeutic strategies. Research on using siRNA for psoriasis treatment has shown good results in blocking different proinflammatory molecules and cytokines. This highlights the potential of this treatment method. Relevant to this investigation, Suzuki et al. [46] created a delivery system for siRNA using hybrid polymer–lipid NPs. They improved this system with photochemical internalization (PCI), using the photosensitizer TPPS2a to help TNF‐α siRNA escape from endosomes into the cytoplasm. This system was designed for a topical treatment for psoriasis. The results showed that combining PLNTPPS2a‐TNF‐α siRNA with PCI significantly reduced TNF‐α levels. Furthermore, Table 1 also summarizes siRNA nanodelivery systems for other autoimmune diseases, including IBD and psoriasis.

## 5. Challenges in Developing RNA‐Based Drugs

### 5.1. Off‐Target Effects

Even with progress, off‐target effects are still a major problem in RNA‐based therapies. siRNAs achieve gene silencing by precisely matching sequences with the target transcript. siRNA can bind not only fully complementary mRNA sequences but also miRNA‐like sites in the 3′ UTR via seed sequences. Such nonspecific binding causes unexpected gene silencing and off‐target effects [47]. Given that these seed sequences are only 6–7 nucleotides in length, the nonspecific target sites they bind to are largely unpredictable and, consequently, difficult to circumvent.

To mitigate such off‐target effects and reduce miRNA‐like off‐target activity, several effective strategies have been developed. First, chemical changes to RNA molecules, like 2′‐O‐methyluridine, can improve specificity. These changes reduce unwanted off‐target interactions similar to those caused by miRNA while still allowing the intended gene silencing to happen [47]. Second, using multiple siRNAs that target the same gene can keep the treatment effective while reducing individual off‐target effects. Also, careful RNA sequence design, especially through detailed bioinformatics analysis to avoid potential off‐target binding sites, can help create more specific RNA therapies. Current research is making progress in developing safer and more effective RNA‐based treatments. We anticipate that future advancements in RNA therapeutics will yield increasingly precise targeting capabilities.

### 5.2. Immunogenicity Issues

As part of the antiviral defense mechanism, the innate immune system identifies exogenous RNAs through specific receptors that detect pathogen‐associated molecular patterns [48]. However, a critical factor influencing the safety and efficacy of RNA therapeutics is their inherent immunogenicity [49]. RNA molecules can activate pattern recognition receptors within the innate immune system, which poses a challenge to their therapeutic application [50]. This immune‐stimulating feature might reduce the effectiveness and safety of RNA therapies used for protein replacement or gene regulation.

To reduce immunogenicity, different methods have been tried to control the immune response to RNA molecules. One way is to add modified nucleosides like 2′‐O‐methyluridine, pseudouridine, and 5‐methylcytidine [51]. These changes greatly reduce the activation of pattern recognition receptors, which lessens the immune response and improves the efficiency of therapeutic mRNA translation. Moreover, adjusting the composition and physical–chemical properties of NPs can lower immunogenicity while ensuring effective RNA delivery. As research advances, it is anticipated that more sophisticated and patient‐friendly RNA therapies will emerge, designed to minimize undesirable immune responses.

## 6. Summary and Future Outlook

The use of siRNA, which targets specific gene sequences for silencing, has emerged as a novel therapeutic strategy for various diseases. However, the inherent instability, poor circulation, and low cellular uptake of free siRNA pose major challenges for its clinical use in treating autoimmune diseases [52]. A large number of basic scientific studies are gradually revealing the potential advantages of nanomedicine, particularly in the field of immune diseases [53]. NPs, as promising drug carriers, offer substantial support for RNAi therapeutics. Nanocarriers protect siRNA from nuclease degradation and rapid renal clearance [54]. They improve the serum stability, cellular uptake, and endosomal escape of siRNA. Furthermore, nanomaterial‐mediated siRNA delivery systems can be integrated with other therapeutic modalities, such as photosensitizers, photothermal agents, chemotherapeutic drugs, and imaging agents. They can also function as photosensitizers or radiosensitizers, highlighting their considerable potential for future development.

Most current studies are still limited to animal models. Animal models cannot fully mimic the complexity, chronicity, and heterogeneity of human autoimmune diseases, so preclinical efficacy often cannot be replicated in patients [55]. With the increasing number of clinical studies on RNAi therapeutics, new challenges remain to be addressed. Firstly, the low transfection efficiency of siRNA nanodelivery vectors, due to the complexity of physiological barriers, limits their potential for clinical applications. Secondly, the synthesis mechanisms of most siRNA nanodelivery vectors are highly complex, which not only complicates the synthesis process but also diminishes the versatility of the method. Furthermore, the synthesis process often necessitates the introduction of additional coupling agents, which frequently lack therapeutic efficacy and may even exhibit toxicity, thereby further complicating the system.

To sum up, the main obstacle in using RNAi‐based treatments is the effective and targeted delivery of siRNAs in clinical settings. Present studies have largely focused on employing siRNAs to treat a narrow spectrum of autoimmune diseases, with RA being the primary target. Future research, including basic experiments, preclinical studies, and clinical trials, should be extended to more common autoimmune diseases. Typical examples include multiple sclerosis and systemic lupus erythematosus. Even with the current limitations and challenges, siRNA‐based treatments offer a new approach for managing autoimmune diseases, holding the potential to transform research findings from the lab into practical clinical solutions. This progress marks the beginning of an exciting new chapter in molecular therapeutics.

## Funding

This work was supported by grants from the Guangdong Basic and Applied Basic Research Foundation (Grant 2025A1515012661), Guangxi Natural Science Foundation (Grant 2024GXNSFAA010096), Shenzhen Science and Technology Program (Grants JCYJ20230807150913027 and JCYJ20240813165111016), Guilin Science Research and Technology Development Project (Grants 20230135‐4‐2 and 20220139‐13‐2), Guangdong Province Medical Science and Technology Research Foundation (Grant B2025207), Guangxi Medical and Health Appropriate Technology Development and Promotion Project (Grant S2024075), National Natural Science Foundation of China (Grants 82460324 and 82101877), Innovation Training Program for College Students (Grants S202510601079, X202510601230, and X202510601258), Guangxi Key Laboratory of Tumor Immunology and Microenvironmental Regulation (Grants 2023KF006, 3030302213, and 2021KF001), Shenzhen Hospital (Longgang) of Beijing University of Chinese Medicine “Elite Talent Cultivation” Postdoctoral Program (Grant 2023‐BUCMSZYLRC42), Chronic Disease Management Research Project of National Health Commission Capacity Building and Continuing Education Center (Grant GWJJMB202510025042), and the Bagui Youth Top‐notch Personnel Program of Guangxi.

## Conflicts of Interest

The authors declare no conflicts of interest.

## Data Availability

The datasets generated and/or analyzed during the study are available from the corresponding author upon reasonable request.
